# Text mining and expert curation to develop a database on psychiatric diseases and their genes

**DOI:** 10.1093/database/bax043

**Published:** 2017-06-26

**Authors:** Alba Gutiérrez-Sacristán, Àlex Bravo, Marta Portero-Tresserra, Olga Valverde, Antonio Armario, M.C. Blanco-Gandía, Adriana Farré, Lierni Fernández-Ibarrondo, Francina Fonseca, Jesús Giraldo, Angela Leis, Anna Mané, M.A. Mayer, Sandra Montagud-Romero, Roser Nadal, Jordi Ortiz, Francisco Javier Pavon, Ezequiel Jesús Perez, Marta Rodríguez-Arias, Antonia Serrano, Marta Torrens, Vincent Warnault, Ferran Sanz, Laura I. Furlong

**Affiliations:** 1 Research Group on Integrative Biomedical Informatics (GRIB), Institut Hospital del Mar d'Investigacions Mèdiques (IMIM), DCEXS, Universitat Pompeu Fabra (UPF), C/Dr. Aiguader 88, Barcelona 08003, Spain; 2 Neurobiology of Behaviour Research Group (GReNeC), Institut Hospital del Mar d'Investigacions Mèdiques (IMIM), DCEXS, Universitat Pompeu Fabra (UPF), Barcelona, Spain; 3 Institut de Neurociències and Animal Physiology Unit, Universitat Autònoma de Barcelona (UAB), Barcelona, Spain; 4 Network Biomedical Research Center on Mental Health (CIBERSAM); 5 Department of Psychobiology, Facultad de Psicología, Universitat de València, València, Spain; 6 Institute of Neuropsychiatry and Addiction, Institut Hospital del Mar d'Investigacions Mèdiques (IMIM), Parc de Salut Mar, Universitat Autònoma de Barcelona (UAB), Bellaterra, Spain; 7 Programa de Cáncer (IMIM), Investigación Traslacional en Neoplasias Colorrectales, C/Dr. Aiguader 88, Barcelona, Spain; 8 Institut de Neurociències and Unitat de Bioestadística, Universitat Autònoma de Barcelona (UAB), Bellaterra, Spain; 9 Institut de Neurociències and Psychobiology Area, Universitat Autònoma de Barcelona (UAB), Bellaterra, Spain; 10 Neuroscience Institute and Department of Biochemistry and Molecular Biology, School of Medicine, Universitat Autònoma de Barcelona (UAB), Bellaterra, Spain; 11 Unidad de Gestión Clínica de Salud Mental, Instituto de Investigación Biomédica de Málaga (IBIMA), Hospital Regional Universitario de Málaga, Málaga, Spain

## Abstract

Psychiatric disorders constitute one of the main causes of disability worldwide. During the past years, considerable research has been conducted on the genetic architecture of such diseases, although little understanding of their etiology has been achieved. The difficulty to access up-to-date, relevant genotype-phenotype information has hampered the application of this wealth of knowledge to translational research and clinical practice in order to improve diagnosis and treatment of psychiatric patients. PsyGeNET (http://www.psygenet.org/) has been developed with the aim of supporting research on the genetic architecture of psychiatric diseases, by providing integrated and structured accessibility to their genotype–phenotype association data, together with analysis and visualization tools. In this article, we describe the protocol developed for the sustainable update of this knowledge resource. It includes the recruitment of a team of domain experts in order to perform the curation of the data extracted by text mining. Annotation guidelines and a web-based annotation tool were developed to support the curators’ tasks. A curation workflow was designed including a pilot phase and two rounds of curation and analysis phases. Negative evidence from the literature on gene–disease associations (GDAs) was taken into account in the curation process. We report the results of the application of this workflow to the curation of GDAs for PsyGeNET, including the analysis of the inter-annotator agreement and suggest this model as a suitable approach for the sustainable development and update of knowledge resources.

**Database URL**: http://www.psygenet.org

**PsyGeNET corpus:** http://www.psygenet.org/ds/PsyGeNET/results/psygenetCorpus.tar

## Introduction

Psychiatric disorders pose a substantial burden to the society with a high impact on morbidity and mortality (1,2). Currently, mental disorders affect 27% of the adult population in Europe (http://www.euro.who.int/en/health-topics/noncommunicable-diseases/mental-health/data-and-statistics). Unraveling the genetic architecture of psychiatric disorders is an active research area, which has led to a large body of literature on the matter (3–5). For several psychiatric disorders, such as mood disorders, schizophrenia, alcohol dependence and anorexia nervosa, among others, the current evidence indicates a polygenic nature (6). Despite the advances achieved in the field in the past 10 years, the large number of publications on the genetics of psychiatric disorders and the lack of suitable tools to efficiently explore this information prevent scientists from leveraging such a large volume of data.

Psychiatric disorders Gene association NETwork (PsyGeNET) (7) has been developed to establish a high-quality resource on psychiatric diseases and their associated genes. The PsyGeNET database has been developed by applying text mining tools to extract information from the scientific literature, which is subsequently validated by experts in psychiatry and neurosciences. In the past few years, text mining approaches have increasingly been adopted to assist the development and curation of knowledge resources (8). The biocuration community has recognized the need to incorporate text mining solutions at different stages of the curation process to support the curators’ tasks. For instance, the Rat Genome Database (9) or the BioGRID interaction database (10) applies a variety of text mining tools to assist their curators’ daily work. In addition, text mining evaluation challenges such as BioCreative have incorporated specific activities to evaluate interactive text mining systems for biocurators (11,12). Thus, in PsyGeNET, we have incorporated text mining tools to assist the curation tasks of the experts.

In PsyGeNET, we consider a gene to be associated with a psychiatric disease either if the gene itself or its products play a role in the disease pathogenesis, or if it is a marker for the disease. In its first release, PsyGeNET focused on mood disorders, in particular, depression and bipolar disorders, and addiction to substances of abuse, such as cocaine and alcohol. The first version of the database resulted from integrating gene–disease association (GDA) data from public resources with data extracted from the literature using text mining, followed by curation by domain experts (7). In this communication, we describe the protocol needed to update and extend the PsyGeNET database. The objectives of this work were to incorporate up-to-date information on the diseases and genes covered in the first release of the database, as well as to extend the scope of the database to other psychiatric diseases. The proposed methodology involves several aspects: (i) the extraction of information of GDAs from the literature using the text mining system BeFree (13); (ii) the recruitment of a team of experts to curate the information extracted by text mining; (iii) the elaboration of a curation workflow, (iv) the development of a web-based annotation tool in order to facilitate the curation task and (v) the definition of detailed guidelines to assist the curation task and the training of the curators. We present the results of the curation process and the analysis of the inter-annotator agreement, describing the main difficulties encountered and the way in which they were addressed. We highlight the importance of recording negative findings from the literature in knowledge resources. Finally, we suggest that this protocol is a suitable approach for the sustainable development and update of curated knowledge resources.

## Materials and Methods

### Curation team

A team of 22 curators from different areas of expertise (such as psychiatry, neuroscience, medicine, psychology and biology) was recruited from the Spanish Network of Addiction (RTA-ISCIII), as well as from the network of collaborators of the coordination team (Research Group on Integrative Biomedical Informatics, GRIB). The incentives for their participation were to become a part of the PsyGeNET team and to be co-authors in the publication(s) originated from the project. The curators were trained at the beginning of the project using the PsyGeNET annotation guidelines as a starting point and then during the pilot phase. Permanent communication with the coordination team by e-mail was established to answer questions during the entire curation process. In addition, online and face-to-face meetings were organized during the two analysis phases, in order to share experiences among the curators and improve the curation pipeline.

### Defining the psychiatric diseases in terms of UMLS concepts

In PsyGeNET, the psychiatric diseases are identified by UMLS Metathesaurus concepts (14). The selection of the psychiatric disorders was based on the interest of our group of curators. The definition of the categories was based on the disease definitions from the Diagnostic and Statistical Manual of Mental Disorders (https://www.psychiatry.org/psychiatrists/practice/dsm) (DSM-5), whereas concepts from the UMLS Metathesaurus were chosen to represent the diseases and to define the eight psychiatric disorders of interest in a standard and public reference vocabulary. In this way, each psychiatric disorder was represented by a set of Concept Unique Identifiers from the Unified Medical Language System (UMLS). The purpose of using the UMLS Metathesaurus in PysGeNET is that the UMLS Metathesaurus integrates different vocabularies and ontologies from both the clinical and research domains, and therefore, it constitutes a very convenient resource for the identification of diseases in the literature due to its comprehensiveness and mapping capabilities. Three domain experts reviewed the terminology of > 2000 UMLS concepts related to the psychiatric disorders of interest, and assigned them to the following psychiatric disease categories (DCs): (i) depressive disorders (*Depression*), (ii) bipolar disorders and related disorders (*Bipolar disorder*), (iii) substance induced depressive disorder (*SI-Depression*), (iv) schizophrenia spectrum and other psychotic disorders (*Schizophrenia*), (v) substance induced psychosis (*SI-Psychosis*), (vi) alcohol use disorders (*Alcohol UD*), (vii) cannabis use disorders (*Cannabis UD*) and (viii) cocaine use disorders (*Cocaine UD*). This information was used both for the text mining of GDAs by BeFree (see below) and for the identification of DCs during the curation process.

### Text mining of gene–disease associations

BeFree (13) was used to identify associations between genes and the psychiatric diseases of interest from a corpus of 1 million of MEDLINE abstracts focused on the genetic basis of human diseases. The corpus was obtained using the Pubmed retrieval system for selecting MEDLINE articles using specific MeSH terms (("Psychiatry and Psychology Category"[Mesh] OR "Diseases Category" [Mesh]) AND "genetics"[Subheading] AND (hasabstract[text] AND ("1980"[PDAT] : "2015"[PDAT]) AND English[lang])). BeFree (13) is a text mining tool based on Natural Language Processing for the identification of biomedical entities and their relationships from scientific publications. It includes two applications for information extraction, namely Named Entity Recognition (NER) and Relation Extraction (RE). The BeFree NER module, a dictionary and rule-based approach, is able to identify diseases and genes and disambiguate them to vocabulary standards. The RE module uses a supervised machine learning approach to detect relationships between entities, such as genes and diseases, by exploiting both shallow and deep syntactic information from the text. The performance of BeFree for identification of associations between genes and diseases has been evaluated in different corpora, achieving competitive results according to the state of the art. For instance, a precision of 84% with a recall of 71% (F-score 76%) was obtained using the EU-ADR corpus as a gold standard (13).

Despite its high performance, an initial evaluation by the text mining developers was performed to identify the most frequent text mining errors of BeFree in a real information extraction scenario. This allowed the identification of ambiguous mentions for genes and diseases such that certain post-processing rules were defined and applied after the NER step in order to address them. For example, the term ‘depression’ was frequently found after the terms ‘cardiac’ or ‘respiratory’, without referring to depression as a psychiatric disease.

For the extraction of information for PsyGeNET, the diseases were identified using the dictionary developed with the terms of the UMLS concepts that define each disorder, whereas an in-house developed gene dictionary was used to identify the genes, as described in (15). The identified disorders were grouped according to the eight psychiatric DCs as described in the previous section. BeFree identified 6349 associations between genes and DCs (gene-disease category associations or GDCAs) at the sentence level, supported by 14 410 publications. Subsequently, several filters were applied to reduce the size of the curation task and make it feasible with the curation resources at hand. We removed review articles and associations already present in curated resources included in the DisGeNET (16) database (CTD_human, CLINVAR, ORPHANET, GWASCAT and UNIPROT) and the previous release of PsyGeNET (7), keeping only those associations published recently (after year 2000) in journals with Science Citation Index (SCI) impact factor >1. The main difference between PsyGeNET and DisGeNET is that all the GDAs present in PsyGeNET have been manually curated by experts, whereas the text mining data present in DisGeNET are not validated by experts. After this process, we keep 2507 GDCAs supported by 2909 publications, which were submitted to expert curation.

### Annotation guidelines

The PsyGeNET annotation guidelines were developed with the purpose of guiding the manual curation process. The guidelines included the definition of a GDA, the way it should be classified according to the association qualifiers and the type of information that should be considered for the annotation. Real examples of the different association qualifiers were provided. Finally, a tutorial on how to use the PsyGeNET annotation tool was also included

The goal of the curation was to validate the association of a gene with a particular disease. We consider that a gene is associated with a disease if the gene itself or the product of the gene plays a role in the disease pathogenesis, or if it constitutes a biomarker for the disease. We did not consider pharmacogenomics studies as evidence for a GDA. The PsyGeNET annotation tool was used to support the curation task. For each GDA identified by text mining, the annotation tool displayed the evidence supporting the association, in specific, the abstracts and the sentences in which the GDA was stated. Then, by inspecting this evidence (both the sentences and the full-text publication, if required), the curator had to qualify the association as *Association*, *No Association*, *False*, *Error* and *Not Clear*. The association qualifiers are described as follows: (i) *Association*: the publication clearly states that there is an association between the gene and the disease—it can be a causative association (e.g. a mutation in the gene causes the disease), or a biomarker association (e.g. an SNP in the gene identified as significantly associated with a disease in a GWAS study); (ii) *No Association*: the publication clearly states that there is no association between the gene and the disease (e.g. a publication that reports a negative finding on the association between the gene and the disease), (iii) *False*: the gene and the disease are found together in the publication, but the study does not address the role of the gene in the disease pathogenesis and (iv) *Error*: when there is a text mining error in the correct identification of the gene and/or the disease. Table 1 shows some examples of the association qualifiers considered in PsyGeNET. In the example for *False*, the variant on the RORA gene was studied for its association with depressive disorder, but not with seasonal affective disorder; therefore, the association between RORA and seasonal affective disorder has to be qualified as *False*. In the example of *Error*, OCT is erroneously identified as a gene, since in the context of the abstract is an acronym of another concept (optical coherence tomography).

**Table 1.** Examples of Association qualifiers. Disease and genes that have to be evaluated are highlighted in the sentence in green and orange, respectively.

Association Type	PMID	Sentence
Association	267012	The **D-amino acid oxidase activator gene (G72)** has been found associated with several psychiatric disorders such as **schizophrenia**, major depression and bipolar disorder
No Association	17692928	There was no association between **TPH-2** gene variants and **MD** in the same population that had shown a strong association with TPH-1
False	25225167	The findings that have gained support indicate that genetic variants of **RORA** (rs2028122) and CRY1 (rs2287161) associate with depressive disorder, those of RORB (rs7022435, rs3750420, rs1157358, rs3903529) and NR1D1 (rs2314339) with bipolar disorder, and those of NPAS2 (rs11541353) and CRY2 (rs10838524) with **seasonal affective disorder** or winter depression
Error	21174530	**OCT** demonstrated loss of foveal **depression** with distortion of the foveal architecture in the macula in all patients

Although the curation of the associations was performed at the abstract level, the curators were asked to review the full text article in those cases where the abstract was not sufficiently clear in order to decide upon an association. The document describing the curation guidelines is available in the PsyGeNET web page (http://www.psygenet.org/ds/PsyGeNET/html/images/PsyGeNETcuration Gui d elines.pdf).

### Annotation tool

A user-friendly web-based tool was developed to assist both the definition of the psychiatric disorders of interest and the curation of GDAs. The tool was designed to support a remote multi-user environment by user and password authentication. Figure 2 shows a screenshot of the tool for the curation of GDCAs. The tool shows the GDCA to be evaluated (in this example the association between the ETNPPL gene and Bipolar Disorders category), and the corresponding publication. The curator has to review the publication and decide if the association of the gene and the DC holds, and assign an association qualifier using the drop-down menu. To aid the curators’ task, the tool displays the terminology for the gene according to standard resources (NCBI Gene, UniProt and HGNC), and highlights the sentences in which BeFree identified the association between the gene and the disease. If required, the curator can access the full text article using the PubMed hyperlink. The curator is also asked to select the sentence that best supports his/her validation decision, if available. This feature allowed us to collect example sentences in order to create a corpus for the development of text mining software [PsyGeNET corpus (http://www.psygenet.org/ds/PsyGeNET/results/psygenetCorpus.tar)]. In addition, the tool allows us to review previous annotations and provides a progress bar that indicates the number of validations and GDCAs curated by the expert vs. the total number assigned. In this paper, we refer to a *validation* to each publication supporting a particular GDCA. Note that each publication can refer to more than one GDCA and that each GDCA can be supported by several publications.

**Figure 1.**
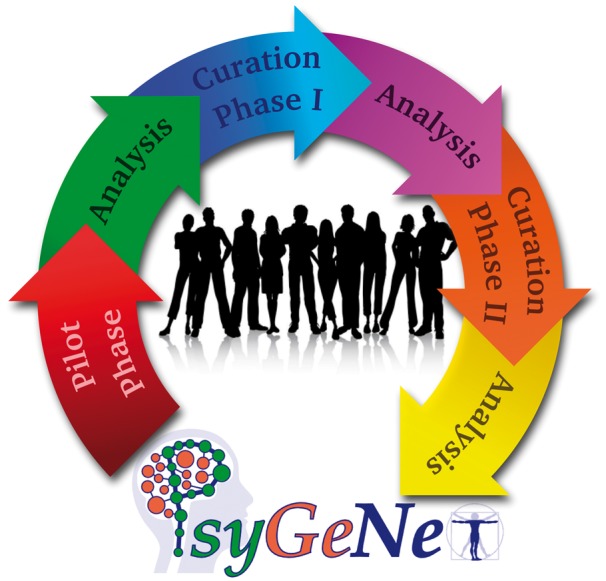
The PsyGeNET curation workflow. The workflow includes: a) a Pilot phase for training of the curators and testing of the annotation tool, b) Curation Phase I and II where the curation of the text-mined data took place, and c) three Analysis phases after each curation to analyze the results and prepare the data for the next stage.

### Curation workflow

We implemented a curation workflow including a pilot phase and two curation and analysis phases (see Figure 1). During the pilot phase, the initial training of the curators was carried out, including how to use the curation tool. A set of 100 publications was validated and analyzed during the pilot phase. After this process both the curation tool and the annotation guidelines were improved based on the received feedback from the experts, and the first curation phase (CP-I) was launched to evaluate 2507 GDCAs identified by text mining and supported by 2909 publications. The results of the curation were analyzed by estimating the inter-annotator agreement at the level of publication. The validations for which a disagreement was found in CP-I (considering any association qualifier) were then reviewed by a third expert during CP-II. Five experts participated in the CP-II. The annotations in which two experts found that the association was *Not Clear* were reviewed by two additional experts in order to assign them to another annotation qualifier, whenever possible. Finally, we included in the database the validations for which agreement of at least two experts was found for the annotation qualifiers *Association* and *No association*.

**Figure 2.**
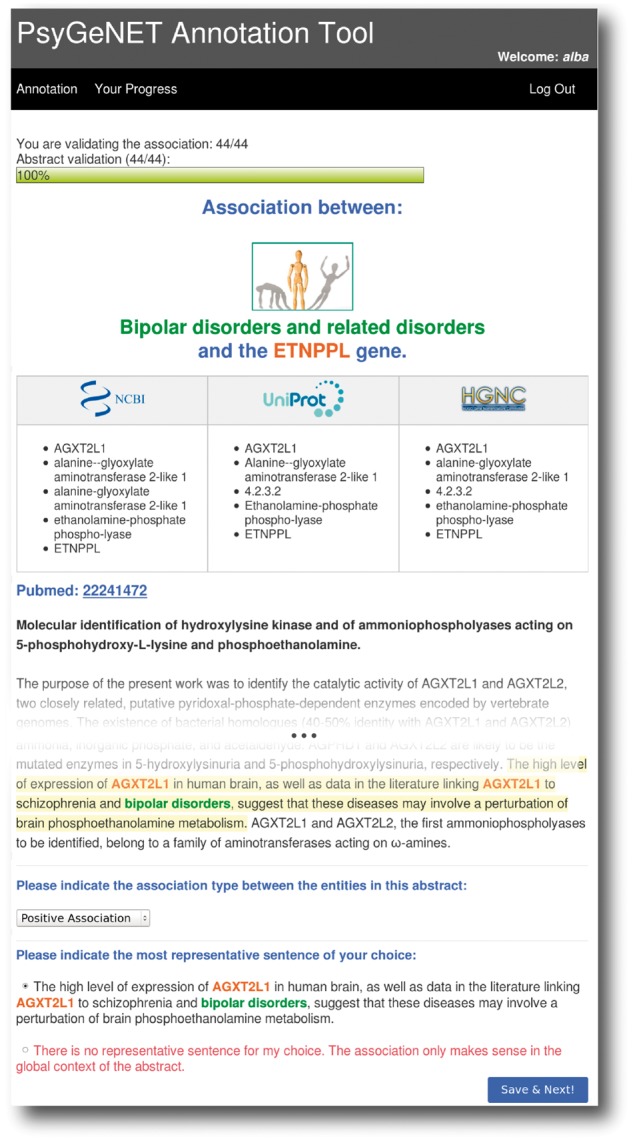
The PsyGeNET annotation tool. A screenshot of the annotation tool is shown, see the text for more details.

## Results and discussion

**Figure 3.**
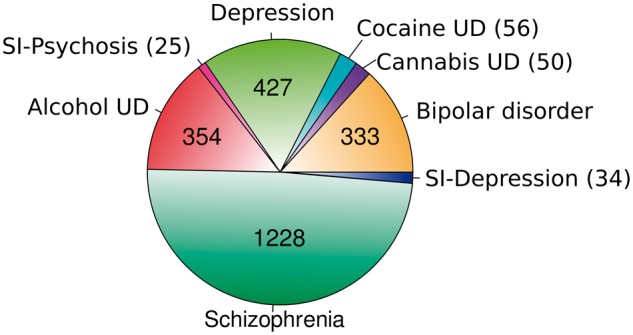
Psychiatric disease categories and the number of associated genes obtained by text mining in the present study, before expert curation.

At the beginning of the process, three experts reviewed the terminology of 2523 UMLS concepts related to the psychiatric disorders of interest. As a result, 1942 UMLS concepts were assigned to one of the eight DCs to be considered in the database, with *Alcohol UD*, *Depression* and *Schizophrenia* being defined as >300 UMLS concepts (321, 368 and 488, respectively). Then, BeFree was used to identify GDAs from the literature based on the above UMLS concepts selection and a set of GDCAs focused on the disorders of interest was identified (see *Text mining of gene-disease association* subsection). The results were filtered out to reduce the size of the curation task and make it more feasible. The 2507 genes identified by BeFree as associated with the DCs were submitted to expert curation. These genes were unevenly distributed across the DCs, with *Schizophrenia* being the DC with more associations followed by *Depression* and *Alcohol UD* (see Figure 3).

Most of the GDCAs were supported by only one publication (70.6%). We included up to five most recent publications for each GDCA for the validation process. This led to 242–284 GDCAs to be validated by each curator, depending on the DC. Since most of GDCAs are supported by only one publication, the number of publications to be reviewed by the curators ranged between 322 and 491. Before starting the curation of 2507 GDCAs, a pilot curation phase was designed with the purpose of training the curators, testing the PsyGeNET annotation tool and fine-tuning the PsyGeNET annotation guidelines. In total, 100 publications were reviewed during the pilot phase and distributed in 10 publications per two experts. The average agreement between the experts’ pairs in the pilot phase was 60%. The main sources of discrepancies were associated with handling speculations and achieving a proper distinction between *False* and *Error* association qualifiers. An *Error* qualifier has to be used when a text mining error, e.g. the incorrect identification of a gene is found in the text. In some cases, due to the large variety of synonyms used in the literature to refer to genes, some experts considered as errors some genes properly detected, thereby leading to annotation discrepancies. For that reason, we improved the explanations in the guidelines by including examples and we modified the tool by adding a table that displayed the terminology for a specific gene according to standard sources (NCBI Gene, UniProt and HGNC). Furthermore, following the experts’ suggestion, the annotation tool was also improved such that it allowed the revision of previous annotations.

Then, the proper curation (CP-I in the workflow in Figure 1) was launched and it was completed in 33 days. During CP-I, 2507 GDCAs supported by 2909 publications were reviewed by the curators. Each expert was assigned with a set of approx. 275 GDCAs (corresponding to 450 publications) according to his/her field of expertise (e.g. *Depression* vs. *Schizophrenia*). Some curators evaluated associations from all DCs, whereas others focused on a single category. The results of the CP-I were analyzed in order to identify agreements and disagreements between the experts. Table 2 shows the number of publications validated by each curator team (composed of two experts) and the agreement achieved. The average agreement between all the experts was 68.95%, higher than that obtained in the pilot phase. For one of the curator teams, the agreement was higher (89%) compared to the rest of the teams. We can attribute this higher agreement to the fact that there was some communication between the two experts permitting them to discuss the curation criteria during the CP-I.

**Table 2.** Inter-annotator agreement during CP-I

Teams	Validations	Agreement	Agreement (%)
Team 1	494	325	65.79
Team 2	319	194	60.89
Team 3	489	342	69.94
Team 4	450	402	89.33
Team 5	492	308	62.60
Team 6	508	341	67.12
Team 7	463	317	68.46
Team 8	516	363	70.35
Team 9	334	221	66.17

We observed that for 30% of the total validated GDCAs, there was certain disagreement between the curators for any association qualifier. The CP-II was aimed at reviewing these associations in which no agreement was found between the two experts in the CP-I. It involved 1252 validations, which were reviewed by a third expert. The results of the CP-II were analyzed in order to identify agreements and disagreements of the third expert with one of the previous two. The agreement in the CP-II was 71% (corresponding to 888 validations), higher than the previous phases.

**Figure 4.**
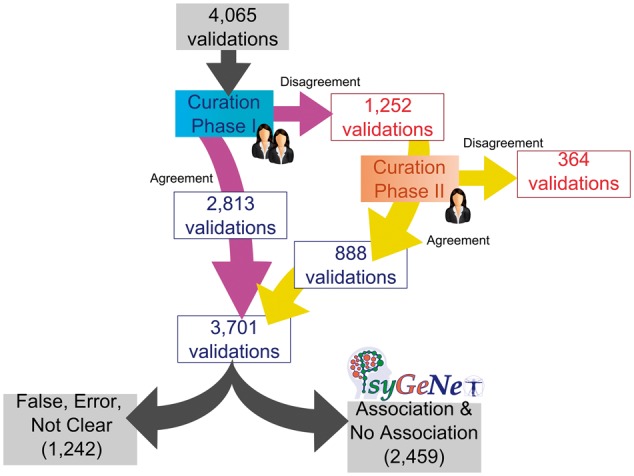
The PsyGeNET curation workflow results. The workflow includes the results in each phase according to the agreement or disagreement between experts and the final number of associations included in the new version of PsyGeNET database (PsyGeNET V.02) according to the evidence that support each annotation.

**Figure 5.**
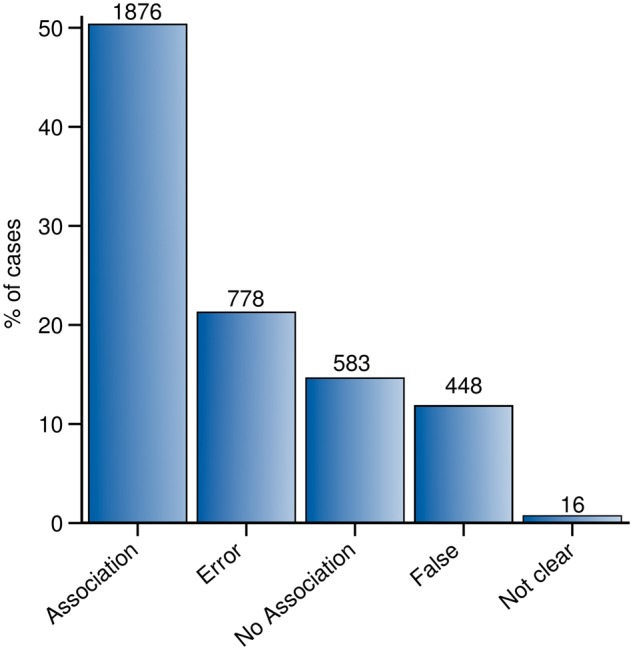
Summary of the agreement results. Each bar in the bar-plot represents the number of validations annotated as: *Association*, *No association*, *False*, *Error* and *Not clear*.

Throughout the whole curation workflow, we found agreement for 91% of the validated GDCAs (Figure 4). Table 3 shows the number of annotations in each curation phase as well as in the entire curation process and the agreement achieved at each step. A substantial fraction of the observed disagreements involved the annotation of an association as *False* by one of the experts (53.28% in CP-I and 75.55% in CP-II). From the 3701 validations in which agreement was found between the experts (2813 validations in CP-I and 888 in CP-II), 2459 were classified as *Association* or *No Association*; 1226 were classified as *False* or *Error*, and only in 16 of them, the evidence extracted from the publication was not sufficient to classify it within any of the previous categories, falling into the *Not Clear* category (Figure 5). The current release of PsyGeNET includes the associations in which at least two experts agreed on the annotation and the association qualifier was *Association* or *No Association* (2459 validations, corresponding to 1606 associations between genes and psychiatric DCs). Notably, an important fraction of these associations (30.45%) contains at least one publication that reports a negative evidence for the GDA. This highlights the importance of recording negative findings from the literature in knowledge resources. In addition, collecting this information is relevant for the development of corpora for the training of text mining systems able to identify negative findings with respect to GDAs from the biomedical literature.

**Table 3.** Number of validations and agreement obtained during each step of the curation process

Curation phase	Total validations	Agreement	Disagreement
CP-I	4065	2813 (69%)	1252 (31%)
CP-II	1252	888 (71%)	364 (29%)
Whole curation workflow	4065	3701 (91%)	364 (9%)

The final results of the curation process were discussed with the experts in order to identify the main difficulties during the annotation in order to improve future curation exercises. The main sources of discrepancies between curators were (i) the difficulty in assessing whether the studies using animal models capture well the disease pathophysiology under investigation, (ii) the consideration of studies focused on pharmacogenomics or response to drug treatments as part of the evidence for a GDA, (iii) studies assessing disease phenotypes (e.g. low mood) in otherwise normal populations and (iv) the assessment of the validity of the statistical analysis in some publications (e.g. GWAS studies). In the first case, the decision on the qualification of the association will depend on the expertise of the curator on animal model research in psychiatry, which was not the same among the team of experts. In the other three cases, the experts expressed difficulties in identifying correctly if an association has to be considered or not. Overall, although the curation task was very focused on the domain of genetics of psychiatric diseases, the wide variety of studies covered by the publications (GWAs studies, sequencing studies, animal models, etc.) requires an equivalent diversity of expertise among the experts. We think that this complexity of the task is one of the main reasons for the inter-annotator agreement achieved in this study. Ongoing work includes revisiting the annotation guidelines to further clarify the curation issues raised, in order to improve the agreement in future annotation exercises.

## Conclusions

In the era of biomedical big data, we present an approach to distill knowledge from the literature by automatic text mining tools coupled to curation by experts in order to enable the development and maintenance of knowledge resources. We designed a protocol that includes curators’ training and the iterative improvement of both the tools and annotation guidelines. We show that engaging with the user community for the curation of the database (in our case the RTA-ISCIII network) proved to be successful for the achievement of the goal of incorporating new information into the database.

Importantly, our curation protocol included the identification of negative findings from the literature. From 1606 associations between genes and psychiatric DCs validated and finally included in the database, 489 of them (30.45%) have at least one negative evidence from the literature. This information has been taken into account for the ranking of the GDA in the new release of PsyGeNET (http://www.psygenet.org/). In addition, the corpus of annotated sentences developed during the curation constitutes a valuable resource for the development and evaluation of text mining systems. This stressed the importance of collecting this information from the literature in a knowledge resource.

## Data availability

All the data generated from this work is publicly available. The document describing the curation guidelines is available in the PsyGeNET web page (http://www.psygenet.org/ds/PsyGeNET/html/images/PsyGeNETcurationGuidelines.pdf). The curated dataset of GDAs is available in the PsyGeNET web portal (http://www.psygenet.org/) and can also be analyzed with the psygenet2r package (https://bioconductor.org/packages/release/bioc/html/psygenet2r.html).  Finally, the  PsyGeNET corpus, consisting of sentences curated by the experts, is also available for the development of text mining tools (http://www.psygenet.org/ds/PsyGeNET/results/psygenet Corpus.tar).

## Funding

We received support from ISCIII-FEDER (PI13/00082, CP10/00524, CPII16/00026), IMI-JU under grants agreements no. 115191 (Open PHACTS)] and no. 115372 (EMIF), resources of which are composed of financial contribution from the EU-FP7 (FP7/2007-2013) and EFPIA companies in kind contribution, and the EU H2020 Programme 2014-2020 under grant agreements no. 634143 (MedBioinformatics) and no. 676559 (Elixir-Excelerate). The Research Programme on Biomedical Informatics (GRIB) is a member of the Spanish National Bioinformatics Institute (INB), PRB2-ISCIII and is supported by grant PT13/0001/0023, of the PE I + D+i 2013-2016, funded by ISCIII and FEDER. MRA, SMR and MCBG are supported RD16/0017/0007; OV, FF and MT are supported by RD16/0017/0010; and AS and FJP are supported by RD16/0017/0001, by Instituto de Salud Carlos III, Red de Trastornos Adictivos (RTA-Retics-ISCIII). AGS acknowledges ﬁnancial support from the Spanish Ministry of Economy and Competitiveness, through the ‘María de Maeztu’ Programme for Units of Excellence in R&D (MDM-2014-0370). Funding for open access: EU H2020 Programme 2014-2020 under grant agreements no. 634143 (MedBioinformatics).

*Conflict of interest*. None declared.
